# Supplementary material for: Statistical multiscale mapping of IDH1, MGMT, and microvascular proliferation in human brain tumors from multiparametric MR and spatially-registered core biopsy
Source: Sci Rep. 2019 Nov 19;9:17112. doi: 10.1038/s41598-019-53256-5 (PMC6864039; doi:10.1038/s41598-019-53256-5)
Supplement: Supplementary file 1 — Supplemental Information [file 41598_2019_53256_MOESM1_ESM.pdf]

Supplementary Information

Title: Statistical multiscale mapping of IDH1, MGMT, and microvascular proliferation in human brain tumors from multiparametric MR and spatially-registered core biopsy

Author Names and Degrees: Jason G Parker*[1,2], PhD, Emily E Diller[2,1], MS, Sha Cao[3], PhD, Jeremy T Nelson[4,1], PhD, Kristen Yeom[5], MD, Chang Ho[1], MD, Robert Lober[6], MD, PhD
*corresponding author

Supplemental Table I. Mean ± standard deviation of sequence parameters across the 5 image contrasts. Parameters that are not specified varied too dramatically across sequence implementation (i.e. 2D vs. 3D read-out) or were not applicable to the sequence.

Parameter	$T1_w$	$T1_{w\text{-}post}$	$T2_w$	T2-FLAIR	DWI
TR (ms)	-	-	3461 ± 955.3	5800 ± 1751.2	10208 ± 4632.9
TE (ms)	4.8 ± 3.4	3.9 ± 2.5	151.9 ± 95.8	330.4 ± 97.8	88 ± 14.3
TI (ms)	-	-	-	1937 ± 294	-
Flip angle (°)	-	-	-	-	99 ± 28.5
Pixel size (mm)	0.8 ± 0.3	0.9 ± 0.2	0.7 ± 0.2	1.0 ± 0.1	1.3 ± 0.3
Bandwidth (Hz/pixel)	173.0 ± 22.1	1088.1 ± 142.3	360.7 ± 252.5	569.8 ± 221.1	1088.1 ± 142.3

Supplemental Table II. Individual predictor results from the binary logistic regression analyses.

Outcome	Predictor	Coefficient	Robust Std. Error	Z	P > |Z|	95% Conf. Interval	
**$IDH1_{MS+}$**	T1wp	0.204	0.140	1.45	0.147	-0.072	0.479
	T1w	-3.211	0.562	-5.71	10^{-4}	-4.313	-2.110
	T2w	1.110	0.036	30.63	10^{-4}	1.039	1.182
	FLAIR	3.673	0.182	20.16	10^{-4}	3.316	4.030
	ADC	-0.459	0.029	-15.89	10^{-4}	-0.516	-0.402
	cons	-11.176	0.487	-22.93	10^{-4}	-12.131	-10.221
**$MGMT_{PMS+}$**	T1wp	2.751	0.132	20.87	10^{-4}	2.493	3.009
	T1w	-4.161	0.175	-23.77	10^{-4}	-4.504	-3.818
	T2w	2.275	0.069	32.76	10^{-4}	2.139	2.411
	FLAIR	0.473	0.195	2.42	0.016	0.090	0.856
	ADC	-4.494	0.237	-18.94	10^{-4}	-4.959	-4.029
	cons	-7.075	0.270	-26.21	10^{-4}	-7.604	-6.546
**$CNEC_{+}$**	T1wp	-0.798	0.020	-39.91	10^{-4}	-0.837	-0.759
	T1w	4.449	0.053	84.29	10^{-4}	4.345	4.552
	T2w	0.305	0.018	17.3	10^{-4}	0.270	0.339
	FLAIR	-2.918	0.030	-98.86	10^{-4}	-2.976	-2.860
	ADC	-1.119	0.015	-77.11	10^{-4}	-1.148	-1.091
	cons	1.936	0.053	36.68	10^{-4}	1.832	2.039
**MVP_{+}**	T1wp	0.049	0.027	1.85	0.065	-0.003	0.102
	T1w	1.175	0.102	11.5	10^{-4}	0.975	1.375
	T2w	0.348	0.024	14.57	10^{-4}	0.301	0.395
	FLAIR	1.492	0.030	50.31	10^{-4}	1.434	1.550
	ADC	0.062	0.052	1.19	0.234	-0.040	0.163
	cons	-7.433	0.104	-71.8	10^{-4}	-7.636	-7.230

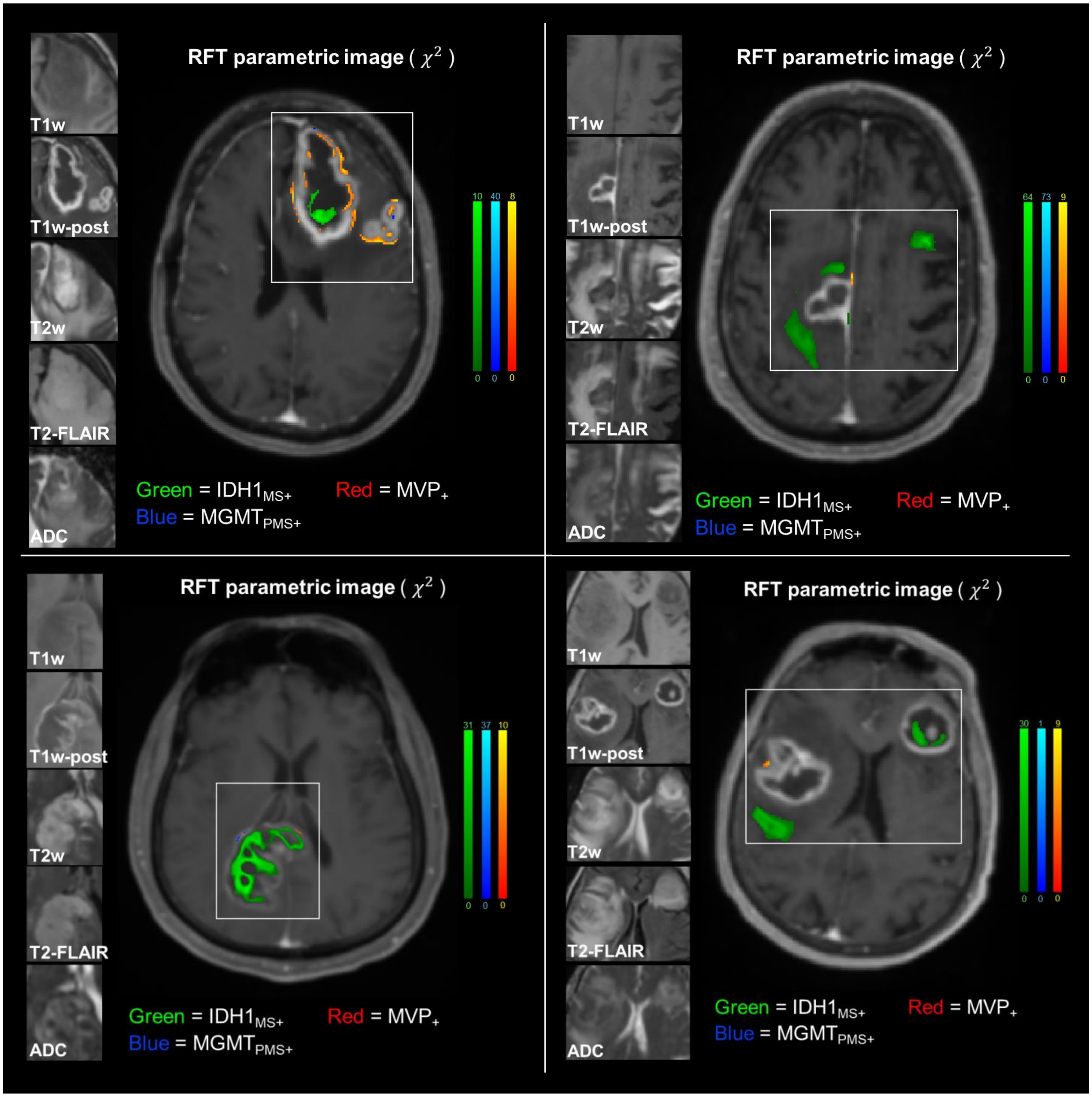

Supplemental Figure I. Statistical parametric maps thresholded by RFT for four additional exemplary patients.
